# Relationship between maximal incremental and high-intensity interval exercise performance in elite athletes

**DOI:** 10.1371/journal.pone.0226313

**Published:** 2020-05-12

**Authors:** Shih-Chieh Chang, Alessandra Adami, Hsin-Chin Lin, Yin-Chou Lin, Carl P. C. Chen, Tieh-Cheng Fu, Chih-Chin Hsu, Shu-Chun Huang

**Affiliations:** 1 Department of Physical Medicine & Rehabilitation, Chang Gung Memorial Hospital, Linkou, Taiwan; 2 Department of Kinesiology, University of Rhode Island, Kingston, RI, United States of America; 3 Department of Physical Medicine & Rehabilitation, Chang Gung Memorial Hospital, Taoyuan branch, Taiwan; 4 College of Medicine, Chang Gung University, Kwei-Shan, Tao-Yuan County, Taiwan; 5 Department of Physical Medicine and Rehabilitation, Chang Gung Memorial Hospital, Keelung, Taiwan; 6 Healthy Aging Research Center, Chang Gung University, Taoyuan City, Taiwan; 7 Department of Physical Medicine and Rehabilitation, New Taipei Municipal Tucheng Hospital, Chang Gung Memorial Hospital, New Taipei, Taiwan; University of Calgary, CANADA

## Abstract

This descriptive study aimed to explore the physiological factors that determine tolerance to exertion during high-intensity interval effort. Forty-seven young women (15–28 years old) were enrolled: 23 athletes from Taiwan national or national reserve teams and 24 moderately active females. Each participant underwent a maximal incremental INC (modified Bruce protocol) cardiopulmonary exercise test on the first day and high-intensity interval testing (HIIT) on the second day, both performed on a treadmill. The HIIT protocol involved alternation between 1-min effort at 120% of the maximal speed, at the same slope reached at the end of the INC, and 1-min rest until volitional exhaustion. Gas exchange, heart rate (HR), and muscle oxygenation at the right vastus lateralis, measured by near-infrared spectroscopy, were continuously recorded. The number of repetitions completed (R_lim_) by each participant was considered the HIIT tolerance index. The results showed a large difference in the R_lim_ (range, 2.6–12.0 repetitions) among the participants. Stepwise linear regression revealed that the variance in the R_lim_ within the cohort was related to the recovery rates of oxygen consumption ($\dot{V}O_{2}$), HR at the second minute after INC, and muscle tissue saturation index at exhaustion (R = 0.644). In addition, age was linearly correlated with R_lim_ (adjusted R = −0.518, p < 0.0001). In conclusion, the recovery rates for $\dot{V}O_{2}$ and HR after the incremental test, and muscle saturation index at exhaustion, were the major physiological factors related to HIIT performance. These findings provide insights into the role of the recovery phase after maximal INC exercise testing. Future research investigating a combination of INC and HIIT testing to determine training-induced performance improvement is warranted.

## Introduction

Currently, incremental exercise testing (INC) is widely used to assess cardiopulmonary fitness among various populations from elite athletes and semi-professional players to chronic cardiovascular and lung disease patients [1–4]. The INC is used to quantify whole-body all-out performance, and for athletes, it has become the gold-standard evaluation to identify exercise intensity zones upon which athletic training programs are designed. Since Taylor et al. [5] showed that the response to INC tests is strongly correlated with the type of protocol followed and the personal characteristics of the individual undergoing the test, much attention has been given to designing optimal standardized INC tests that account for fitness abilities and testing goals (e.g., determination of ventilatory thresholds, maximal oxygen consumption ($\dot{V}O_{2\max}$), etc.) [4]. In this context, Muscat et al. [6] recommended the use of a ramped increase in work rate to obtain a reliable evaluation of mode-specific fitness to determine the most favorable training protocols.

In many competitive ball games, including soccer and basketball, and games such as badminton, a typical mode-specific task is represented by the combination of repeated efforts and recovery phases that closely replicate actual field-match performance. At high professional levels, the intensity of this performance can be compared to high-intensity interval workouts. High-intensity interval training (HIIT), a very popular training methodology, can be described as a workout strategy alternating between short-duration high-intensity efforts and passive or active recovery periods. Independent of the format of the training protocol (i.e., intensity, duration, number of bouts, and sets), HIIT has several practical advantages that make it appealing to ball sports players, such as the possibility of improving rapidly (e.g., to shorten the preparation periods) and maintaining optimal fitness for a longer time period due to a faster recovery capacity [7]. The main goals for using HIIT as a preferred training method for professional players are to quickly improve whole-body aerobic capacity; to reduce delay between mechanical requests (i.e., exercise tasks) and muscle metabolic response; to shorten the recovery time between repeated efforts, rounds, and matches; and to reduce the onset of fatigue. These performance improvements are related to a series of aerobic and anaerobic adaptations induced by HIIT, such as an increase in skeletal muscle mitochondria dimensions, higher blood pH tolerance, and increased anaerobic capacities [7–12], among others.

Even though HIIT has been widely used in practice, it is seldom used as a testing protocol [13, 14], with INC being used to plan the training calendar. Very few studies with small groups of soccer players have used intermittent running tests (e.g., YO-YO intermittent recovery test) to determine changes in performance after the players followed HIIT training [15–18]. To date, none have considered the relationship between INC and HIIT protocols in evaluating the performance of professional team players.

In 2015–2016, a group of reserve athletes from the Taiwan national soccer, basketball, and badminton teams visited our laboratory at the Chang Gung Memorial Hospital for a series of routine performance evaluations. During that time, we administered a traditional INC protocol and HIIT. We set the HIIT exercise intensity at 120% of the highest velocity reached during the INC, and we considered the number of the repetitions completed (R_lim_) as the tolerance index for each participant. The main aim of this descriptive study was to determine which factors are strongly associated with the limitations in HIIT. We hypothesized that R_lim_ in HIIT varies greatly among individuals despite the same relative intensity and exercise-recovery duration and pattern, which we suggest is primarily due to individual recovery capacities.

## Materials and methods

### Participants

All participants provided written informed consent after receiving oral and written explanations of the experimental procedures and associated risks. This research was performed in accordance with the ethical standards of the Declaration of Helsinki. This study enrolled 47 athletes and moderately active (MA) female participants. The athletes were reserves for the national Taiwan soccer, basketball, and badminton teams. The participants assigned to the MA group were young women who participated in moderate-intensity exercises for at least 60 min weekly [19]. The experiment protocol was approved by the Chang Gung Memorial Hospital Institutional Review Board.

### Protocol

The participants visited our laboratory at the Chang Gung Memorial Hospital twice to perform an INC maximal test and HIIT. Each participant was instructed to refrain from vigorous exercise or caffeine intake for 24 h prior to testing and to have at least 8 h of sleep the night before the tests. All assessments took place approximately at the same time of the day under controlled environmental conditions (24°C, 63% humidity).

#### Anthropometric and body composition evaluations

At the beginning of the visit, basic anthropometric characteristics (height and weight) were recorded. Subsequently, whole-body compositions were determined using the InBody s10 analyzer (Seoul, Korea) and by measuring electrical resistance to four different frequencies (5, 50, 250, and 500 kHz) [20–22]. Each participant lay on a padded table for the entire duration of the testing and the sensors to measure electrical resistance were placed at the level of each body segment as per the manufacturer’s instructions. The participants were instructed to fast for 2 h prior to the test, which was 20 min long.

#### Cardiopulmonary testing

During the first visit, maximal INC test was performed. The test started with 1 min of walking at a rate of 1 mile/h, followed by an incremental modified Bruce protocol conducted until voluntary exhaustion (i.e., subject failed to keep up with the treadmill speed despite strong encouragement) was reached. The INC was defined as maximal when the following criteria were met: (i) no further increase in $\dot{V}O_{2}$ between the two stages sustained for at least 2 min despite strong encouragement ($\dot{V}O_{2}$ plateau was attained), (ii) heart rate (HR) exceeds 85% of its predicted maximum, and (iii) the respiratory exchange ratio exceeds 1.15 [2]. Immediately after exhaustion, an active recovery phase at the individual’s walking pace was administered for 1 min, followed by 3 min of passive recovery.

During the second visit that took place at least 24 h after the first visit, a supra-maximal HIIT test was performed. The speed was set at 120% of the highest velocity reached during the INC, and the slope was the same as the INC final stage. At the beginning of the visit, the participants became familiar with the protocol by simulating one repetition at 120% of the highest velocity attained by the individual during INC, and then recovery. After a 10-min rest, the HIIT testing began with 1 min of walking at a rate of 1 mile/h, followed by intermittent 1-min sprinting interspersed with 1-min passive recovery until volitional exhaustion. The total number of repetitions completed before exhaustion was recorded to define the R_lim_ for each individual.

### Measurements

All exercise assessments were performed on an electromechanically braked treadmill (VIASYS^™^) that was connected to and operated by a metabolic cart (MasterScreen CPX, CareFusion, Hoechberg, Germany) that also allowed continuous, breath-by-breath measurements of pulmonary minute ventilation (${\dot{V}}_{E}$) and mouth gas exchange (i.e., $\dot{V}O_{2}$ and rate of carbon dioxide production [$\dot{V}CO_{2}$]). Before each test, the gas analyzers and the turbine flow meter of the system were calibrated as per the manufacturer’s instructions, using a gas mixture of known concentrations (FO_2_: 0.16; FCO_2_: 0.05; N_2_ as balance) and an automatic high and low pumping flow system. Heart rate was determined from the R-R interval on a 12-lead electrocardiogram.

Muscle oxygenation was evaluated by means of a portable continuous-wave and spatially resolved near-infrared spectroscope (NIRS) with Bluetooth (PortaMon, Artinis, the Netherlands). Relative concentrations of deoxyhemoglobin+myoglobin (HHb) and oxyhemoglobin+myoglobin (O_2_Hb) and the tissue saturation index (TSI, %) [23] were continuously recorded during the exercises at the level of the peripheral muscle tissue, 1.5 cm beneath the probe (interoptode distance: 3 cm). From these measurements, the relative changes in total hemoglobin and myoglobin (THb = HHb + O_2_Hb) were calculated. The NIRS probe was wrapped in plastic foil and placed longitudinally to the vastus lateralis belly on the right thigh, 15 cm above the upper margin of the patella, and secured with an elastic band to minimize the possibility of external light influencing the signal. The sampling rate was set at 1 Hz.

### Data analysis

The individual $\dot{V}O_{2\max}$ was determined by analyzing the INC breath-by-breath gas exchange data. If a plateau was present (increase of <2 ml·kg^−1^·min^−1^ despite an increased workload [24, 25]), the $\dot{V}O_{2\max}$ was calculated by averaging the final 30 s recorded before exhaustion; otherwise, the highest $\dot{V}O_{2}$ value recorded within the last 30 seconds of test was retained as the individual $\dot{V}O_{2\max}$ [26]. Maximal HR was defined as the highest HR value in the breath-by-breath data.

For the recovery phase after INC, a five-breath moving average was applied to the $\dot{V}O_{2}$ data [27], previously interpolated to 1-sec intervals [28]. The data at time point 30, 60, and 120 s were chosen for further analysis. For the NIRS data (i.e., TSI and O_2_Hb), the 1-Hz raw data were used to calculate the time points at 30, 60, and 120 s, and t_1/2_ in the recovery phase.

The$\dot{V}O_{2}$, HR, O_2_Hb, and TSI changes at 0.5 min recovery after INC were calculated, as suggested by Turner et al. [29]:

$$\Delta \dot{V}O_{2}0.5=\dot{V}O_{2max}-\dot{V}O_{2}\;at\;30s\;in\;the\;recovery\;phase$$ Eq 1

$$\Delta \dot{V}O_{2}0.5/=\Delta \dot{V}O_{2}0.5/\dot{V}O_{2max}.$$ Eq 2

**Fig 1:**
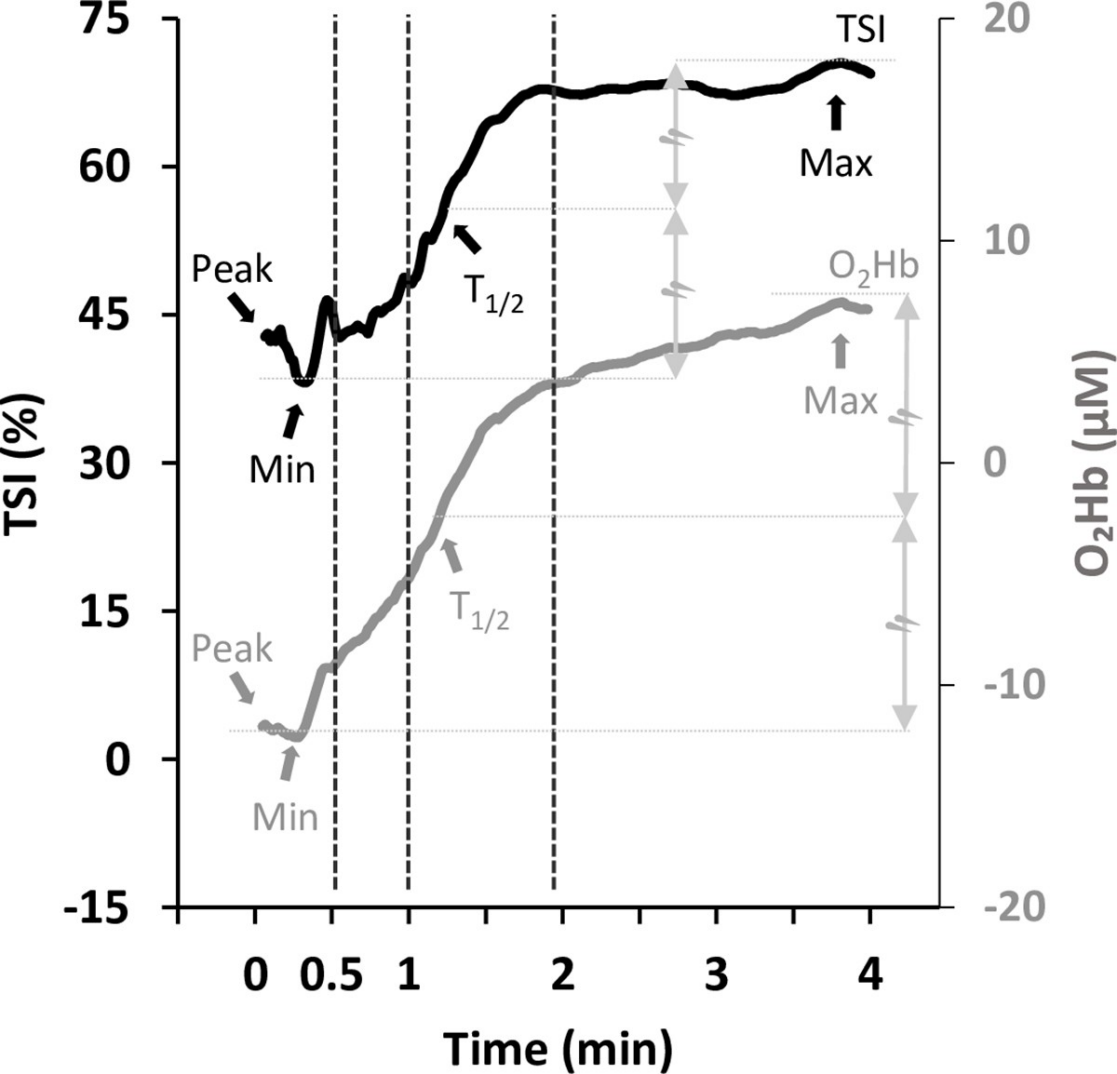
NIRS-derived tissue saturation index (TSI, black continuous trace) and oxygenated-(hemoglobin+myoglobin) (O_2_Hb, grey continuous trace) responses during the recovery phase of incremental (INC) exercise tests. Color-coded arrows indicate the maximum (*Max*) and minimum (*Min*) values for both parameters reached during recovery; *Peak* indicates the value attained at the end of the incremental test (at min zero, 0, on the x-axis).

The above equations were also applied to determine the deltas (i.e., Eq 1) and the change ratio (i.e., Eq 2) at the first and second minute, for which rate of $\dot{V}O_{2}$ was the O_2_ uptake measured at 60- and 120-s recovery, respectively. The same approach was used for calculating HR and the NIRS-derived variables (TSI, O_2_Hb), for the same time points during the recovery phase of the INC test. In addition, TSIrt_1/2_, O_2_Hbrt_1/2_, $\dot{V}O_{2}rt_{1/2}$, and HRrt_1/2_ are the time spans required for the values to achieve half of their recovery to the baseline value (*Min* to *Max*) (Fig 1).

### Statistical analysis

Data are presented as means and standard deviations (SDs). Statistical significance was set at *p* <0.05. Pearson’s, Spearman’s, and partial correlations were used to determine the degree of association between anthropometric and physiological variables versus R_lim_. Since the population sample size allocated to the final analysis was 47, the first five parameters with the greatest correlation coefficients were included in the regression model. Forward stepwise linear regression was performed to identify the R_lim_ predictors. Analyses were performed using SPSS 22.0 (SPSS, Inc., Chicago, IL, USA).

## Results

**Fig 2:**
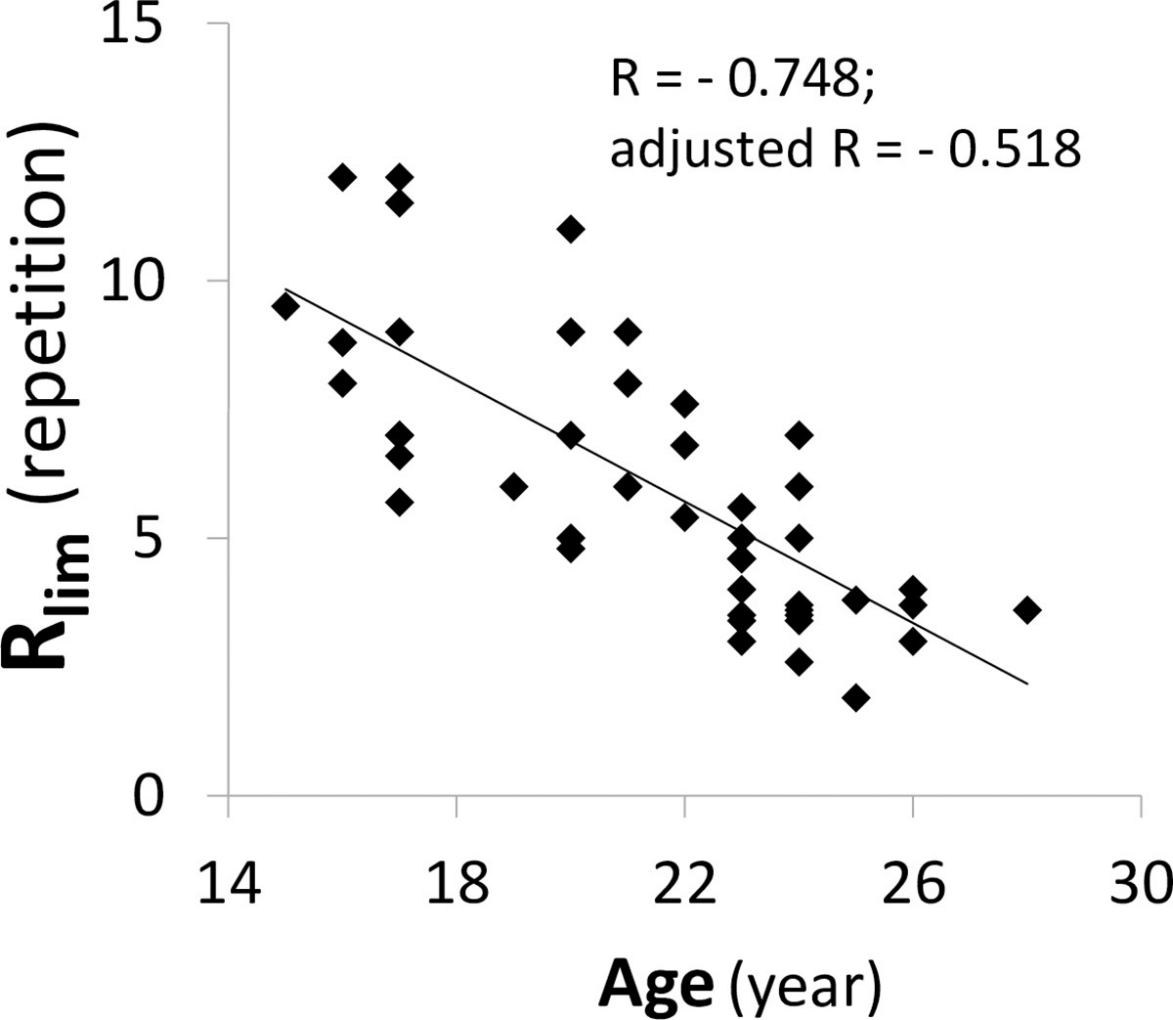
**Scatter plots showing the relationship between the total number of high-intensity interval test repetitions and age (Panel A).** A partial correlation was utilized to adjust the variables with co-linearity, including group and percent body fat (PBF).

**Fig 3:**
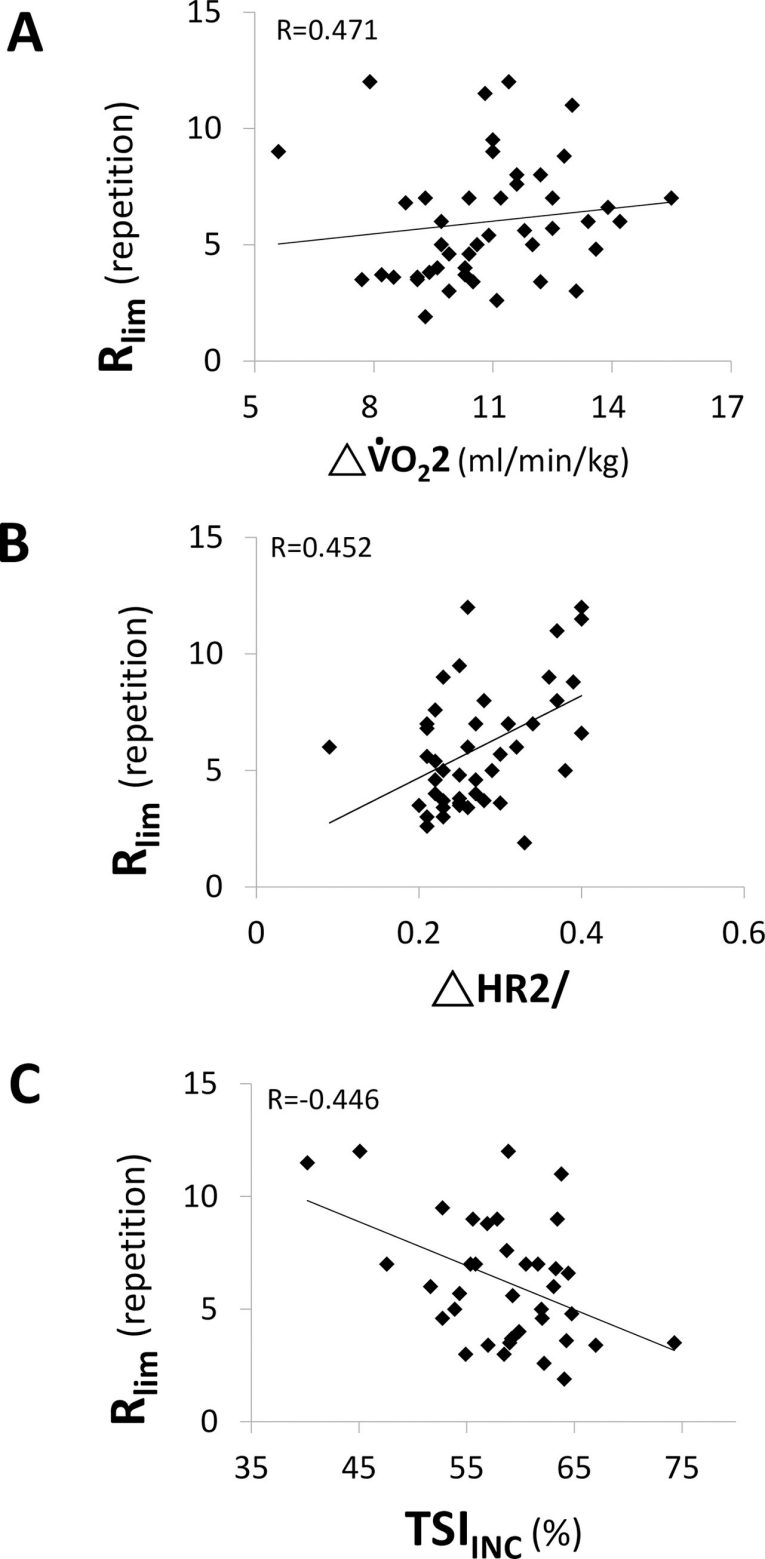
Scatter plots showing the relationship between the total number of high-intensity interval test repetitions and $\Delta \dot{V}O_{2}2$ (= $\dot{V}O_{2\max}$ − $\dot{V}O_{2}$ at 2 min during recovery; **Panel A**), ΔHR2/ (= (HR_max_ − HR at 2 min during recovery)/maximal HR; **Panel B**) or TSI_INC_ (tissue saturation index at exhaustion; **Panel C**), determined by incremental exercise test data.

Forty-seven participants (n = 23 athletes; n = 24 MA) successfully completed the experimental protocol phases. All the participants reached a $\dot{V}O_{2}$ plateau at the end of the INC test. The R_lim_ differed greatly among participants and ranged from 2.6 to 12.0 repetitions. Pearson’s or Spearman’s correlations showed that R_lim_ was significantly correlated with age (r = -0.748), group (r = 0.74), and percent body fat ([PBF] r = 0.371). Age was still significantly correlated with R_lim_ (R = -0.518; *p* < 0.0001) after adjusting variables with co-linearity, including group and PBF in partial correlations (Fig 2). The PBF did not correlate with R_lim_ after adjusting for age and group (R = -0.194, *p* = 0.201). When the HIIT and INC were compared by univariate analyses, several physiological parameters during INC were significantly correlated with R_lim_. Those parameters with a correlation coefficient >0.3 and p-values <0.05 are shown in S1. The five physiological parameters with the highest correlation coefficients ($\Delta \dot{V}O_{2}2$, $\dot{V}O_{2\max}$, ΔHR2/, TSI_INC,_ and ΔHR2) were evaluated in the forward linear stepwise regression model (Table 1). The explanatory power in model 3 of multiple linear regression was 0.415. It revealed that R_lim_ was primarily determined by $\Delta \dot{V}O_{2}2$, ΔHR2/, and TSI_INC_. Their scatter plots to R_lim_ are shown in Fig 3. Correlations were positive in $\Delta \dot{V}O_{2}2$ and ΔHR2/ and negative in TSI_INC_ (Fig 3A, 3B and 3C).

**Table 1:** Stepwise linear regression results on R_lim_ predictors.

	ß	t	P(ß)	R	ΔR^2^	F
Model 1				0.471	0.222	9.396*
$\Delta \dot{V}O_{2}2$	0.471	3.065	0.04			
Model 2				0.572	0.106	7.798*
$\Delta \dot{V}O_{2}2$	0.368	2.419	0.021			
**ΔHR2/**	0.342	2.247	0.032			
Model 3				0.644	0.086	7.304*
$\Delta \dot{V}O_{2}2$	0.282	1.884	0.069			
**ΔHR2/**	0.317	2.192	0.036			
**TSI**_**peak**_	-0.309	-2.139	0.040			

*p *<*0.05; the p-value indicates the overall significance of the linear regression model

P(ß): p-value for ß

The main INC and HIIT findings are presented in Table 2. As expected, the athletes had significantly higher $\dot{V}O_{2\max}$, maximal O_2_ pulse, peak ${\dot{V}}_{E}$, lower TSI_INC_, and better performance in HIIT (R_lim_: 7.8 ± 2.3 vs. 4.2 ± 1.4, *p* < 0.05) compared to the MA participants.

**Table 2:** Average values of physiological parameters for the athlete and MA groups in INC and HIIT tests.

	Ath	MA
**INC**		
$\dot{V}O_{2\max}$ (mL.min^-1^.Kg^-1^)	45.7 ± 6.0*	37.8 ± 7.6
peak HR (bpm)	185.0 ± 8.4	187.4 ± 10.0
peak O_2_ pulse (mL per beat)	12.8 ± 1.8*	10.4 ± 1.9
EqCO_2_ nadir	24.3 ± 2.2	24.1 ± 2.2
EqO_2_ nadir	20.4 ± 2.1	21.1 ± 2.7
peak V_E_ (mL.min^-1^)	83.0 ± 13.5*	76.3 ± 14.0
V_E_/VCO_2_ slope	26.8 ± 3.3	25.4 ± 6.9
TSI_INC_ (%)	56.4 ± 6.6*	61.0 ± 5.1
HHb_peak_ (μM)	0.9 ± 5.0	2.1 ± 5.0
O_2_Hb_peak_ (μM)	-4.1 ± 4.7	-6.6 ± 4.5
THb_peak_ (μM)	-3.3 ± 6.5	-4.5 ± 7.9
**HIIT**		
R_lim_ (repetition)	7.8 ± 2.3*	4.2 ± 1.4

Values are shown as mean ± SD

* *p* <0.05 in Mann-Whitney U test; INC: maximal incremental exercise testing; HIIT: high-intensity interval testing; Ath: athletes; MA: moderately active participants; HR: heart rate; V_E_: ventilation; TSI: tissue saturation index; HHb: deoxyhemoglobin; O_2_Hb: oxyhemoglobin; THb: total hemoglobin; TSI_peak_: nadir of tissue saturation index during INC; EqCO_2_: ventilator equivalent for CO_2_; EqO_2_: ventilatory equivalent for O_2_

The main results in the recovery phase of INC are presented in Table 3. Heart rate and $\dot{V}O_{2}$ recovery were faster in the athletes than in the MA participants. Muscle oxygenation recovery showed no difference between groups; while ΔHR2/ and $\Delta \dot{V}O_{2}2$, the major determinants of R_lim_ according to the stepwise regression (Table 1), were significantly higher in athletes than in the MA participants.

**Table 3:** Comparison of physiologic response of athlete and MA groups during the recovery phase at the end of maximal incremental exercise testing.

	Ath	MA		Ath	MA		Ath	MA		Ath	MA
ΔHR0.5	9.4 ± 6.3	8.8 ± 3.5	$\Delta \dot{V}O_{2}0.5$	14.1 ± 5.9*	8.8 ± 3.5	ΔTSI0.5	3.52 ± 3.42	3.01 ± 2.50	ΔO_2_Hb0.5	3.73 ± 2.52	3.05 ± 2.33
ΔHR1	24.2 ± 9.0	20.1 ± 6.3	$\Delta \dot{V}O_{2}1$	23.8 ± 4.3*	20.1 ± 6.3	ΔTSI1.0	8.19 ± 4.42	7.35 ± 3.55	ΔO_2_Hb1.0	8.24 ± 3.05	7.72 ± 4.00
ΔHR2	56.5 ± 11.8*	45.5 ± 10.1	$\Delta \dot{V}O_{2}2$	34.6 ± 5.5*	27.6 ± 6.8	ΔTSI2.0	12.73 ± 6.47	11.23 ± 2.93	ΔO_2_Hb2.0	11.82 ± 3.37	10.94 ± 4.53
ΔHR0.5/	0.05 ± 0.03	0.05 ± 0.02	$\Delta \dot{V}O_{2}0.5$/	0.3 ± 0.1	0.05 ± 0.02	ΔTSI0.5/	0.23 ± 0.21	0.21 ± 1.16	ΔO_2_Hb0.5/	0.27 ± 0.17	0.20 ± 0.13
ΔHR1/	0.13 ± 0.05	0.11 ± 0.03	$\Delta \dot{V}O_{2}1$/	0.5 ± 0.1	0.11 ± 0.03	ΔTSI1.0/	0.57 ± 0.23	0.51 ± 0.21	ΔO_2_Hb1.0/	0.59 ± 0.16	0.50 ± 0.19
ΔHR2/	0.31 ± 0.06*	0.24 ± 0.06	$\Delta \dot{V}O_{2}2$/	0.7 ± 0.0	0.24 ± 0.06	ΔTSI2.0/	0.84 ± 0.16	0.77 ± 0.15	ΔO_2_Hb2.0/	0.83 ± 0.12	0.70 ± 0.19
HRr_t1/2_ (s)	84.9 ± 18.6	92.4 ± 12.2	$\dot{V}O_{2rt1/2}$ (s)	53.5 ± 8.6	92.4 ± 12.2	TSI_t1/2_ (s)	53.1 ± 14.1	29.3 ± 14.1	O_2_Hb_t1/2_ (s)	59.5 ± 23.7	49.0 ± 15.6

Data are mean ± SD

* Ath vs. MA, *p* <0.05 in Mann-Whitney U test

Ath: athletes; MA: moderately active participants

ΔHR0.5' (min^-1^) = peak HR − HR at 0.5 min recovery; ΔHR1 (min^-1^) = peak HR − HR at 1 min recovery; ΔHR2(min^-1^) = peak HR − HR at 2 min recovery; ΔHR0.5/ = ΔHR0.5/ maximal HR; ΔHR1/ = ΔHR1/ maximal HR; ΔHR2/ = ΔHR2/maximal

The above equations also applied to $\dot{V}O_{2}$ (mL.min^-1^.Kg^-1^)

ΔTSI0.5 (μM) = TSI at 0.5 min recovery—peak TSI; ΔTSI1 (μM) = TSI at 1 min recovery—peak TSI; ΔTSI2 (μM) = TSI at 2 min recovery—peak TSI; ΔTSI0.5/ = ΔTSI0.5/ (maximal TSI–minimal TSI) recovery; ΔTSI1 / = ΔTSI1/ (maximal TSI–minimal TSI) recovery; ΔTSI2/ = ΔTSI2/ (maximal TSI–minimal TSI) recovery

The above equations also applied to O_2_Hb (μM)

Anthropometric data showed no differences in body weight, body height, and body mass index (BMI); however, age was significantly different between groups (Ath vs. MA = 19 ± 3 vs. 24 ± 2 years; *p* < 0.05) between athletes and MA participants. Table 4 shows the body composition data. Athletes had higher soft lean mass (SLM), skeletal muscle mass (SMM), segmental muscle mass at right arm (SMRA), segmental muscle mass at right leg (SMRL), segmental muscle mass at trunk (SMTR), fat-free mass (FFM), protein, body cell mass (BCM), and lower PBF than the MA group. The segmental muscle mass at left arm (SMLA) and segmental muscle mass at left leg (SMLL) p-values were 0.051 and 0.055, respectively.

**Table 4:** Body composition characteristics of participants.

	Ath	MA
SMM (Kg)	23.6 ± 2.3*	22.2 ± 2.3
Fat (Kg)	11.8 ± 2.7	13.7 ± 3.8
PBF (%)	21.5 ± 4.1*	24.9 ± 4.5
SLM (Kg)	40.4 ± 3.7*	38.3 ± 3.7
FFM (Kg)	42.9 ± 3.9*	40.6 ± 3.9
SMRA (Kg)	2.1 ± 0.3*	1.9 ± 0.3
SMLA (Kg)	2.0 ± 0.3	1.9 ± 0.3
SMTR (Kg)	18.8 ± 1.8*	17.9 ± 1.7
SMRL (Kg)	6.9 ± 0.8*	6.5 ± 0.8
SMLL (Kg)	6.9 ± 0.7	6.6 ± 0.8
Protein (Kg)	8.5 ± 0.8*	8.0 ± 0.8
BCM (Kg)	28.0 ± 2.6*	26.6 ± 2.6
TBM/FFM (%)	73.3 ± 0.2	73.3 ± 0.2

Value are shown as Mean ± SD

* *p* <0.05 in Mann-Whitney U test; Ath: athletes; MA: moderately active subjects; SMM: Skeletal Muscle Mass; PBF: percent body fat; SLM: Soft Lean Mass; FFM: Fat Free Mass; SMRA: segmental muscle right arm; SMLA: segmental muscle left arm; SMTR: segmental muscle trunk; SMRL: segmental muscle right leg; SMLL: segmental muscle left leg; BCM: body cell mass; TBM/FFM: total body mass/fat free mass

## Discussion

This descriptive study aimed to investigate the relationship between the ability to sustain HIIT and fitness level, determined by a modified Bruce incremental protocol. We considered the number of HIIT repetitions completed as the tolerance index for each participant (i.e., the higher number of HIIT repetitions completed, the higher the effort tolerance). Our results showed that the total number of repetitions needed to reach the participant’s limitation (R_lim_) was widely distributed among the study population, ranging from 2.6 to 12.0 repetitions (mean: 6.0 ± 2.6 repetitions). Linear regression analyses showed that HR recovery, $\dot{V}O_{2}$ recovery, and TSI_INC_ were the main variables determining the ability to perform HIIT for long durations (i.e., higher number of repetitions completed).

### Parameters derived from INC influencing R_lim_

**Fig 4:**
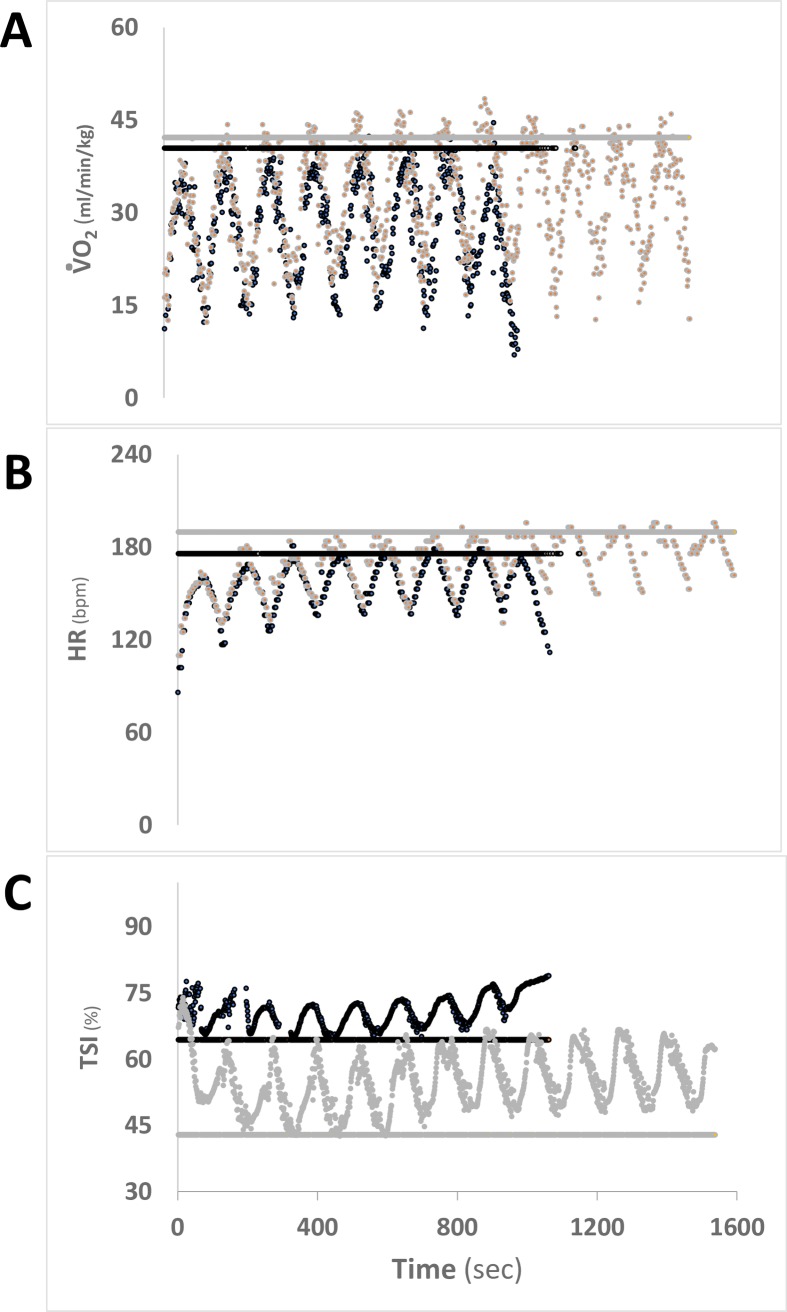
HIIT physiological responses from two representative participants are reported for: $\Delta \dot{V}O_{2}$ (**Panel A**), HR (**Panel B**), and TSI (**Panel C**). Example of a participant able to complete a higher number of HIIT repetitions (in grey); dark traces represent a subject with lower tolerance to HIIT. Horizontal continuous lines represent the maximal values for the respective physiological parameters reached at the end of the INC test. HIIT: high-intensity interval testing, INC: maximal incremental exercise test, $\dot{V}O_{2}$: oxygen consumption, TSI_INC_: nadir of tissue saturation index during INC.

Traditionally, INC tests have been performed to obtain a physiological quantification (e.g., $\dot{V}O_{2\max}$) reflecting the whole-body cardiopulmonary fitness of an individual. Maximal $\dot{V}O_{2}$, the main parameter determined by INC testing procedures, and anaerobic threshold are used to describe the ability of the cardiopulmonary system to integrate with metabolic and central nervous system activities [3]. In this study, participants may reach similar $\dot{V}O_{2\max}$ values at the end of the INC test (Fig 4A), but they significantly differed in the number of repetitions completed at the end of HIIT (see example of two representative subjects that completed 12.0 vs. 7.7 repetitions, respectively; Fig 4). To determine which physiological parameters can explain the large R_lim_ variance in this cohort, linear regression analyses were performed.

Recovery of $\dot{V}O_{2}$ ($\Delta \dot{V}O_{2}2$), rather than $\dot{V}O_{2\max}$, was found to be the strongest determinant of the R_lim_. This result is in agreement with a previous study by Harris et al., [30] who investigated the time course of phosphocreatine (PCr) re-synthesis in a group of adults undergoing a maximal test performed using a cycle ergometer. These authors observed that muscle recovery kinetics after exhaustive maximal exercise was biphasic with the alactacid component ranging from 10 s to a few minutes and the lactacid component lasting a few minutes to hours [31, 32]. The alactacid component consists of oxygen-dependent adenosine triphosphate (ATP) replenished with PCr, which is the primary energy replenishment pathway during HIIT. Participants with faster ATP/PCr replenishment have more rapid $\dot{V}O_{2}$ recovery within the first minutes after dynamic exhaustive exercise and thereby have a broader $\dot{V}O_{2}$ reserve for the next HIIT repetition. As evidenced by our statistical model, these individuals are characterized by the presence of high R_lim_.

The second parameter that appears to determine the performance capacity during HIIT is the HR recovery (ΔHR2/). This reflects the regulatory capability of cardiac autonomic nervous systems [33]. Previous studies showed that HR recovery is related to $\dot{V}O_{2\max}$ [34–36] and training status [37, 38]. The present study further found that it is also related to performance in HIIT. During HIIT, faster HR recovery suggests the presence of a broader HR reserve for the next repetition. This highlights the role of cardiac autonomic nervous system regulation in determining HIIT performance.

Besides the recovery rate for $\dot{V}O_{2}$ and HR, TSI_INC_ was found to be the third significant parameter to explain the variance in R_lim_ among the study participants. Tissue saturation index represents a balance between muscular oxygen delivery and consumption [39, 40]. Increase in TSI accompanied by an increase in mitochondrial biogenesis, capillarization, and mitochondrial enzyme activity, have been shown after HIIT training [41–43]. It is likely that participants who reached a lower TSI_INC_ at the end of the INC protocol had higher peripheral muscular metabolic acidosis tolerance and, thus, a greater capacity to sustain HIIT efforts, resulting in higher R_lim_.

In addition, R_lim_ and age showed a strong relationship among the young-adult participants that volunteered to take part in this study (age range: 15–28 years). This finding seems to suggest that the capacity to sustain long HIIT durations is related to the maturity (age) of the individual undergoing the test. Ratel et al. [44] compared the recovery capacity of prepubescent boys (n = 11; age, 9.6 ± 0.7 years), pubescent boys (n = 9; age, 15 ± 0.7 years), and adult men (n = 10; age, 20.4 ± 0.8 years) using a ten-repetition intermittent sprinting cycling test (friction load = 50% optimal force), separated by 30-s, 1-min, and 5-min passive recovery durations. The capacity of maintaining peak cycling power from the first to the 10^th^ set decreased (p < 0.01) by 11.3% in adult men, 15.3% in pubescent boys, and no changes were noted in the prepubescent group, when the recovery interval was 1 min (the same duration applied in the current study for the HIIT test). Their findings [44] suggest that for the pubescent and adult categories, a longer recovery time was needed due to higher muscle glycolytic activity and slower PCr re-synthesis. Similarly, in a study by Zafeiridis et al. [45], the effect of age was investigated with respect to the capacity of recovery after high-intensity intermittent isokinetic strength exercises. Groups of boys (age, 11.4 ± 0.5 years), teens (age, 14.7 ± 0.4 years), and men (age, 24.1 ± 2 years) were enrolled and each group performed two sets of exercises of 30-s and 60-s bout durations, separated by 1- and 2-min rest periods, respectively. The results showed that the teens tended to recover faster than men, suggesting that the rate of recovery for both types of tasks was age related. Accordingly, the recovery capacity from anaerobic performance decreases with age, with the decline starting as early as 9 to 11 years. Age-related exercise capacity decline is multi-factorial, involving elements such as decrease in intramuscular PCr and intramuscular creatine kinase concentrations, rates of PCr hydrolysis, and glycolytic enzymatic activities as well as changes in muscle architecture and speed of neural activation [31, 46–50]. Moreover, younger adolescents rely less on anaerobic glycolysis and more on aerobic metabolism than older adolescents [51, 52]. Consequently, the former are likely to experience less fatigue and recover more quickly than the latter in HIIT testing. However, due to the wide age range and exercise types (aerobic or anaerobic) [53–55], the mechanisms underlying the age-related decline in anaerobic performance from teens to young adults remains unclear.

The parameters of HR, $\dot{V}O_{2}$ recovery, and TSI at the end of INC showed an explanatory power of 0.415 (Model 3, Table 1). This suggests that additional factors should be considered to explain the variance in R_lim_ seen in our cohort. One plausible factor might be the participants’ motivation and compliance. As recently reported by Noakes et al. [56, 57], although aerobic fitness and the central nervous system play major roles, athletes’ biological condition at the exercise onset (e.g. emotional state like motivational self-belief; mental and physical fatigue) affects in part the performance of an individual.

### Limitations

This study has a few limitations. First, our findings might apply to HIIT protocols similar to the one used in this study, but they might not be consistent for other HIIT protocols where a different work/recovery format (e.g., repetition duration, intensity) is set or if the tolerance is evaluated with field-type tests.

Second, it could seem rather surprising that the values of HR and $\dot{V}O_{2}$ during the second minute of recovery after INC (i.e., $\Delta \dot{V}O_{2}2$, ΔHR2/) showed a stronger correlation with R_lim_ than those measured immediately after the end of the same test (i.e., at 30 and 60 seconds). These findings can be partially explained by the difference existing between active and passive recovery. During the recovery phase, all participants were instructed to walk at their own comfortable speed at 0% grade for 1 min (active recovery) and to stand still for an additional 2 min (passive recovery). However, the HRs decreased less in active recovery than in passive recovery, where the latter has a lower central command from the motor cortex and muscle mechano-metabo receptor activity from skeletal muscle contractions [58]. The recovery $\dot{V}O_{2}$ is also higher in active than in passive recovery [59]. The competition for oxygen between PCr replenishment and muscle activity during active recovery produces a higher $\dot{V}O_{2}$ [60, 61]. In the first minute of active recovery, the recovery kinetics were not comparable among the participants.

Thirdly, limitations exist in the use of NIRS methodology in estimating muscle metabolism. High melanin content and large adipose tissue thickness (ATT) can cause signal attenuation, reducing the amount of light reaching the muscle tissue under investigation. Nevertheless, low melanin content (all the participants were of Asian ethnicity) and an average ATT below 1.6–1.8 mm for the whole cohort, confirmed the reliability of our NIRS data [62].

Furthermore, the participants included in our study were all female. Although only a few studies in the literature have included a similar large number of women as in this study, the influence of sex on the results of our analyses deserves further investigation. While menstrual cycles and oral contraceptives were not controlled or documented at the time of testing, evidence is available showing that neither hormonal fluctuations between the menstrual cycles nor low-dose oral contraceptives used by athletes today have detectable effects on maximal exercise performance [63].

Finally, for this study, the intensity of HIIT exercises was calculated as a fixed percentage of the highest speed reached at the end of the INC test. The use of an INC-derived fixed percentage to calculate the HIIT work rate should be considered another determinant of the variability in R_lim_ results in this context. In 2019, Iannetta et al. [64] reported that when work rates are set as fixed-percentage of parameters measured at maximal exercise capacity, given the between-subjects variability in percentages defining the intensity of an exercise domain (i.e., moderate, heavy, severe, extreme [65]), an increased variance in metabolic stimulus/response is expected. In our study, this correlates to metabolic stress and unequal relative work seen in the HIIT participants’ responses (e.g., oxygen uptake ranged between 82–91% and 95.4–112% of the individual $\dot{V}O_{2\max}$ during the first and following HIIT repetitions, respectively; Fig 4A). To ensure accurate control of exercise intensity among participants and reduce errors in comparisons, future studies on the physiological response at specific exercise intensity domains should adopt methodological approaches that consider individual physiological thresholds and exercise-intensity domain models as those proposed by Iannetta and colleagues [64].

## Conclusion

The capacity to sustain a HIIT exercise, determined by the number of repetitions completed before exhaustion (i.e., R_lim_) differs greatly among young-adult female athletes. The recovery rates of $\dot{V}O_{2}$ and HR after incremental testing and peripheral muscle saturation levels at exhaustion in INC are the major physiological factors related to R_lim_. Moreover, age represents an additional R_lim_-influencing factor.

These findings provide insights into the importance of the recovery phase after maximal INC exercise testing. The values derived from the recovery phase specifically predict HIIT performance and possibly predict sport performance where repeated high-intensity efforts are required. Based on our findings, future research on using a combination of INC and HIIT testing to determine the performance improvement of athletes after HIIT training is warranted.

## Supporting information

S1 Table Pearson correlation coefficients between B_lim_ and cardio-respiratory variables derived from INC for the whole group of participants. (DOCX)

## Abbreviations

Ath: athletes
ATT: adipose tissue thickness
BCM: body cell mass
EqCO_2_: ventilatory equivalent for CO_2_
EqO_2_: ventilatory equivalent for O_2_
FFM: fat free mass
HHb: deoxy-(hemoglobin+myoglobin)
HR: heart rate
HR_max_: maximal HR
HR reserve: heart rate reserve
ΔHR0.5: HR_max_ − HR at 0.5 min recovery
ΔHR1: HR_max_ − HR at 1 min recovery
ΔHR2: HR_max_ − HR at 2 min recovery
ΔHR0.5/: ΔHR0.5/HR_max_
ΔHR1/: ΔHR1/HR_max_
ΔHR2/: ΔHR2/HR_max_
HRrt_1/2_: time span the heart rate recovers to half
HIIT: high-intensity interval testing
INC: maximal incremental exercise test
MA: moderately active participants
Max O_2_ pulse: maximal $\dot{V}O_{2}$/HR
NIRS: near-infrared spectroscopy
O_2_Hb: oxy-(hemoglobin+myoglobin)
ΔO_2_Hb0.5: O_2_Hb at 0.5 min during recovery − peak O_2_Hb
ΔO_2_Hb1: O_2_Hb at 1 min during recovery − peak O_2_Hb
ΔO2Hb2: O_2_Hb at 2 min during recovery − peak O_2_Hb
ΔO_2_Hb0.5/: ΔO_2_Hb0.5/(maximal O_2_Hb − minimal O_2_Hb) during recovery
ΔO_2_Hb1 /: ΔO_2_Hb1/(maximal O_2_Hb − minimal O_2_Hb) during recovery
ΔO_2_Hb2/: ΔO_2_Hb2/(maximal O_2_Hb − minimal O_2_Hb) during recovery
PBF: percent body fat
PCr: phosphocreatine
R_lim_: number of total repetitions to limitation (i.e., exhaustion) in high-intensity interval exercise test
SLM: soft lean mass
SMM: skeletal muscle mass
SMLA: segmental muscle mass, left arm
SMLL: segmental muscle mass, left leg
SMRA: segmental muscle mass, right arm
SMRL: segmental muscle mass, right leg
SMTR: segmental muscle mass, trunk
TBM/FFM: total body mass/fat free mass
T_1/2_: the time span the variable changes in half
THb: total-(hemoglobin+myoglobin)
TSI: tissue saturation index
TSI_INC_: tissue saturation index at exhaustion (immediately before recovery) during INC
ΔTSI0.5: TSI at 0.5 min during INC recovery − peak TSI
ΔTSI1: TSI at 1 min during INC recovery − peak TSI
ΔTSI2: TSI at 2 min during INC recovery − peak TSI
ΔTSI0.5/: ΔTSI0.5/(maximal TSI − minimal TSI) during recovery
ΔTSI1 /: ΔTSI1/(maximal TSI − minimal TSI) during recovery
ΔTSI2/: ΔTSI2/(maximal TSI − minimal TSI) during recovery
$\dot{V}CO_{2}$: rate of carbon dioxide production
${\dot{V}}_{E}$: minute ventilation
$\dot{V}O_{2}$: rate of oxygen consumption
$\dot{V}O_{2\max}$: maximal $\dot{V}O_{2}$
$\Delta \dot{V}O_{2}0.5$: $\dot{V}O_{2\max}$ − $\dot{V}O_{2}$ at 0.5 min recovery
$\Delta \dot{V}O_{2}1$: $\dot{V}O_{2\max}$ − $\dot{V}O_{2}$ at 1 min recovery
$\Delta \dot{V}O_{2}2$: $\dot{V}O_{2\max}$ − $\dot{V}O_{2}$ at 2 min recovery
$\Delta \dot{V}O_{2}0.5$/: $\Delta \dot{V}O_{2}0.5$/maximal $\dot{V}O_{2}$
$\Delta \dot{V}O_{2}1$/: $\Delta \dot{V}O_{2}1$/maximal $\dot{V}O_{2}$
$\Delta \dot{V}O_{2}2$/: $\Delta \dot{V}O_{2}2$/maximal $\dot{V}O_{2}$
/maximal $\dot{V}O_{2}rt_{1/2}$: time span the $\dot{V}O_{2}$ recovers to half
