# Supplementary material for: Relationship between maximal incremental and high-intensity interval exercise performance in elite athletes
Source: PLoS One. 2020 May 12;15(5):e0226313. doi: 10.1371/journal.pone.0226313 (PMC7217474; doi:10.1371/journal.pone.0226313)
Supplement: S1 Table — (DOCX) [file pone.0226313.s001.docx]

**Table S1.** Pearson correlation coefficients between B_lim_ and cardio-respiratory variables derived from INC for the whole group of participants

|  | **Δ**V**O_2_2** | V**O_2max_** | **ΔHR2/** | **TSI_INC_** | **ΔHR2** | **HR reserve** | **Δ**V**O_2_2/** | **Δ**V**O_2_0.5** | **Resting HR** | **max O_2_ pulse** |
| --- | --- | --- | --- | --- | --- | --- | --- | --- | --- | --- |
| **B_lim_** (n) | 0.471 | 0.454 | 0.452 | -0.446 | 0.443 | 0.368 | 0.353 | 0.334 | -0.325 | 0.320 |

△VO_2_2 = peak VO_2_ − VO_2_ at 2 min recovery; VO_2max_: maximal VO_2_; △HR2/ = △HR2 /maximal HR; △HR2 = peak HR − HR at 2 min recovery; TSI_peak_: nadir of tissue saturation index during INC; HR reserve: heart rate reserve; △VO_2_2/ = △VO_2_2 / maximal VO_2_; △VO_2_0.5 = peak VO_2_ − VO_2_ at 0.5 min recovery, Resting HR = resting heart rate; Max O_2_ pulse = maximal VO_2_/HR
